# Perceived helicopter parenting and its association with coping skills and stress appraisals in Turkish youth athletes

**DOI:** 10.3389/fpsyg.2025.1630822

**Published:** 2025-10-16

**Authors:** Tuba Denizci, Rabia Hurrem Ozdurak Singin, Hakan Kaya, Laurentiu-Gabriel Talaghir, Teodora Mihaela Iconomescu, Cristina Corina Bentea

**Affiliations:** ^1^Department of Sport Sciences, Institute of Graduate Studies, Hitit University, Çorum, Türkiye; ^2^Department of Exercise and Sport Sciences, Faculty of Health Sciences, Malatya Turgut Özal University, Malatya, Türkiye; ^3^Faculty of Physical Education and Sports, “Dunărea de Jos” University of Galati, Galaţi, Romania; ^4^Department of Teacher Training, “Dunărea de Jos” University of Galati, Galaţi, Romania

**Keywords:** helicopter parenting, youth athletes, athletic coping skills, challenge and threat appraisals, sports psychology, parental influence

## Abstract

**Introduction:**

Helicopter parenting (HP), characterized by overinvolved behaviors, may impact young athletes’ psychological skills. This study aimed to examine the relationship between perceived HP, athletic coping skills, and stress appraisals (challenge and threat) in Turkish competitive youth athletes aged 13–15 years.

**Methods:**

A total of 398 competitive youth athletes participated, with 302 categorized into HP (*n* = 51) or regular parenting (RP, *n* = 251) groups. The Perceived Helicopter Parenting Scale, Athletic Coping Skills Inventory-28, and Challenge and Threat in Sport Scale were administered at two time points: during a training session and within 30 minutes before a competitive event. Mann-Whitney *U*-tests and repeated-measures ANOVA were used for analysis.

**Results:**

Athletes with HP parents reported significantly lower total coping skills (*p* = 0.001) and lower scores on all subscales except coachability (*p* < 0.05) compared to those with RP parents. Before competitions, HP athletes exhibited lower challenge (*p* < 0.001) and higher threat appraisals (*p* = 0.001), with no differences observed during training.

**Discussion:**

Perceived HP is associated with reduced coping skills and heightened threat appraisals in competitive contexts, highlighting the need for interventions to promote balanced parental involvement in youth sports.

## Highlights

- Perceived helicopter parenting is associated with lower athletic coping skills in Turkish youth athletes.
- Athletes with helicopter parents report higher threat and lower challenge appraisals before competitions.
- Maternal helicopter parenting is more prevalent than paternal in Turkish youth sports.
- Cultural context may influence the impact of helicopter parenting on athletes’ psychological skills.

## 1 Introduction

The holistic approach of sports psychology recognizes that the mind and body are interconnected in sports performance. While physical talent and technical abilities are often prioritized, sports psychology underscores the critical role of sport specific psychological skills, such as mental toughness and stress management, in athletic performance (Weinberg and Gould, 2023). For youth athletes aged 13–15, a developmental stage marked by heightened autonomy needs and sensitivity to parental influence (Steinberg, 2008), these skills are essential for success in competitive settings (Krane and Williams, 2006). Focusing on this age range is essential, as it aligns with the developmental demands of mastering sport-specific psychological skills and navigating social influences, such as parenting styles, that impact athletic outcomes (Krane and Williams, 2006).

This study is grounded in two theoretical frameworks: self-determination theory (SDT) and the biopsychosocial model of stress. Drawing on SDT, which posits that psychological wellbeing hinges on satisfying autonomy, competence and relatedness or feeling connected to others (Deci and Ryan, 2000), helicopter parenting (HP) emerges as a critical construct due to its autonomy-thwarting effects. Unlike authoritative parenting, which balances support and moderate control to foster autonomous regulation, HP defined by excessive control and developmentally inappropriate interventions such as making decisions for the athlete or constantly correcting mistakes, restricts young athletes’ ability to develop independent coping strategies, weakening autonomous motivation and increasing anxiety (Padilla-Walker and Nelson, 2012; Baumrind, 1971). Beyond sports, HP is linked to reduced problem-solving skills and increased emotional distress, amplifying its relevance for youth athletes’ psychological development (LeMoyne and Buchanan, 2011). These effects may also indirectly impair competence and relatedness, further hindering psychological growth in youth sports.

The biopsychosocial model of stress (Engel, 1980) complements SDT by explaining how social factors, including parental involvement, shape athletes’ stress appraisals. These appraisals are categorized as challenge states when perceiving demands as opportunities or threat states when perceiving demands as overwhelming, which influence performance through cognitive and physiological responses (Blascovich and Mendes, 2000; Gürbüz et al., 2021). HP’s overcontrolling behaviors are hypothesized to exacerbate threat appraisals, particularly in high-stress competitive contexts, by diminishing athletes’ perceived control. By integrating these frameworks, HP is positioned as a focal construct, linking SDT’s emphasis on autonomy-driven motivation with the biopsychosocial model’s focus on social influences on context-dependent stress responses, particularly in Türkiye’s family-centric culture where maternal HP is prevalent (Yılmaz, 2020).

Accordingly, the theoretical frameworks guided not only the conceptual focus on HP but also the methodological choices, including scale selection and measurement timing. This conceptual integration guided the study design, with SDT informing the focus on HP’s autonomy-thwarting effects, leading to the selection of the Perceived Helicopter Parenting Attitudes Scale (PHAS; Yılmaz, 2019) to assess overcontrolling behaviors of parents. Unlike scales measuring broader parental behaviors such as Parental Autonomy Support Scale, PHAS isolates HP’s impact on autonomy, aligning with the study’s objectives and ensuring cultural relevance in Türkiye (Yılmaz, 2020). Regular parenting (RP), characterized by balanced support, serves as a control group (Holt et al., 2008).

The biopsychosocial model informed the selection of the Challenge and Threat in Sport Scale (CTSS; Gürbüz et al., 2021) and the dual-timepoint design, measuring CTSS during training where stress is low, and within 30 min before competition where stress level is high, to capture how HP amplifies threat appraisals in high-stakes settings (Blascovich and Mendes, 2000).

Athletic coping skills, defined as cognitive and behavioral strategies to manage stress, such as coping with adversity, maintaining concentration, and regulating emotions (Smith et al., 1995; Özcan and Günay, 2017), are critical for navigating these stress appraisals in both training and competitive contexts. On the other hand, parental involvement significantly shapes athletes’ psychological development. Positive parental support, defined as encouragement, emotional backing, and fostering autonomy without excessive control, enhances athletes’ enjoyment, perceived competence, and self-determined motivation (Holt et al., 2008; Sánchez-Miguel et al., 2013). Research shows that children’s perceptions of parental attitudes and behaviors are more influential than parent-reported behaviors in shaping self-perceptions, affect, and motivation (Horn and Horn, 2007; Brustad, 1996; Wuerth et al., 2004). For instance, positive athlete perceptions of their relationships with parents are associated with greater enjoyment, perceived competence, and lower stress (Sánchez-Miguel et al., 2013). Parental responsiveness, a key component of positive support, involves understanding, validating, and caring for the athlete’s needs and goals, contributing to the wellbeing of both the individual and the relationship (Reis et al., 2004; Reis and Gable, 2015). In contrast, excessive involvement, such as HP, characterized by overcontrol, excessive problem-solving, and developmentally inappropriate tactics, may hinder psychological development by limiting autonomy and fostering dependency (LeMoyne and Buchanan, 2011; Segrin et al., 2012).

HP can undermine athletes’ ability to develop self-efficacy and problem-solving skills, leading to increased anxiety and reduced coping capacity (Padilla-Walker and Nelson, 2012; Rousseau and Scharf, 2017). In Türkiye, cultural norms emphasizing strong familial involvement, particularly maternal caregiving, amplify HP’s autonomy-thwarting effects, necessitating a culturally specific investigation (Yam and Kumcağız, 2021). Recent studies highlight the complexity of parental influence in youth sports, moving beyond a simplistic support-versus-pressure dichotomy (Dorsch et al., 2016). For example, Dorsch et al. (2016) emphasize the dynamic, bidirectional nature of parent-athlete relationships, where the quality of involvement rather than quantity shapes psychological outcomes. Authoritative parenting, characterized by high support and moderate control, fosters positive psychological outcomes, such as autonomous regulation and intrinsic motivation (Holt et al., 2008; Sánchez-Miguel et al., 2013). In contrast, HP, often marked by excessive concern, planning, and perfectionist attitudes, may exacerbate threat appraisals in competitive settings by undermining athletes’ sense of control (Pepe et al., 2025; Yılmaz, 2019). This is particularly relevant in non-Western contexts, such as Türkiye, where cultural expectations may amplify HP’s impact (Yılmaz, 2020).

By examining perceived HP’s effects on athletic coping skills and stress appraisals among Turkish youth athletes aged 13–15, this study hypothesizes that (a) athletes with HP parents will exhibit lower coping skills than those with RP parents, and (b) HP will increase threat and reduce challenge appraisals before competitions. This investigation contributes to sport psychology by elucidating HP’s psychological impact in a culturally relevant setting, offering insights for tailored parental involvement strategies.

## 2 Materials and methods

### 2.1 Study design and sampling

This cross-sectional, quantitative study was approved by Hitit University Non-Interventional Research Ethics Committee on 2 December 2022 (Approval No: 2022-26). The population comprised competitive youth athletes aged 13–15 in Turkey participating in various sports during the 2022–2023 academic year. A power analysis using G*Power assumed a medium effect size (f = 0.25) based on prior studies on parenting and coping skills (Dorsch et al., 2016), with 80% power and α = 0.05, yielding a minimum sample size of 200. To account for potential data loss, ensure sufficient representation of HP, and enable subgroup analyses by parenting style, a larger sample of 612 athletes was randomly selected from the official list of the Provincial Directorate of Youth and Sports. A stratified sampling approach was employed to select athletes competing in various regional and national competitions, ensuring representation across diverse competitive contexts.

### 2.2 Sampling procedure

**FIGURE 1:**
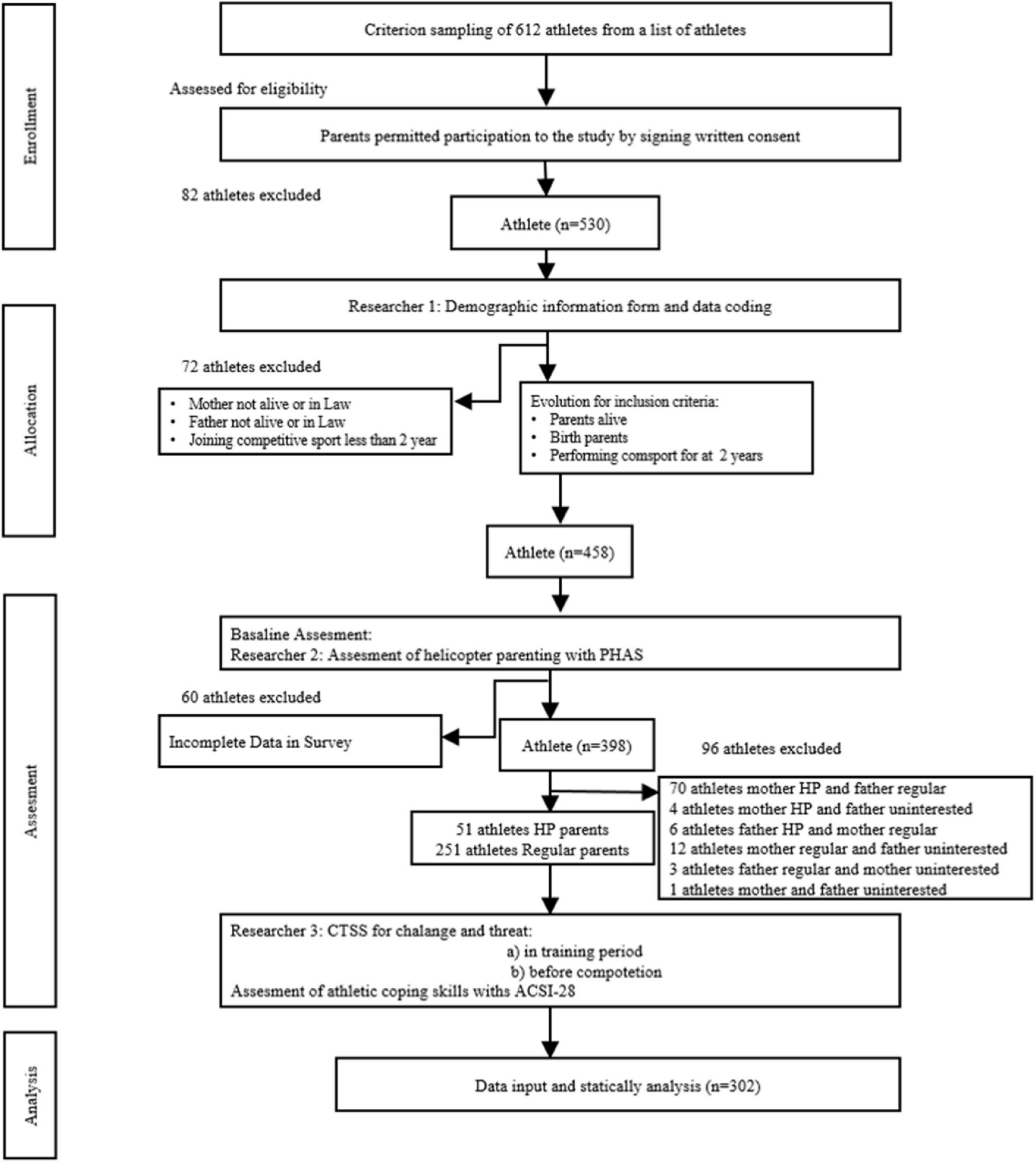
Flowchart depicting a study process with athletes. Starts with 612 athletes; exclusions occur for various reasons, narrowing to 302. Stages include enrollment, allocation, assessment, and analysis. Exclusions cite factors like parental consent and survey completion. Final analysis involves assessing coping skills with ACSI-28.

Athletes were invited through letters sent to their parents, explaining the study’s objectives and obtaining written informed consent. Participants completed an anonymous personal information form using self-selected nicknames to ensure confidentiality. Of 530 respondents, 72 were excluded due to having non-biological or deceased parents, and 60 were excluded for incomplete surveys, resulting in 398 eligible athletes. For analysis, 302 athletes were grouped as having both parents with HP (*n* = 51) or RP (*n* = 251) parenting attitudes, based on the Perceived Helicopter Parenting Attitudes Scale (Yılmaz, 2019) (Figure 1). Cases with mixed parenting, where one parent showed HP and the other RP, or uninterested parenting attitudes were excluded to ensure clear group comparisons and avoid confounding effects from inconsistent parental influences, as mixed or uninterested attitudes could obscure the relationship between parenting style and psychological outcomes. Data were collected at two distinct time points to capture psychological responses under different stress levels, aligning with the biopsychosocial model’s emphasis on context-dependent stress responses (Engel, 1980). During a training session which is a non-stressful context, athletes completed the PHAS, ACSI-28 and CTSS to establish baseline measures of parenting perceptions, coping skills, and stress appraisals in a quiet room to ensure a controlled and distraction-free environment. Within 30 min before a competitive event for stressful context, only the CTSS was reapplied to assess challenge and threat appraisals under heightened performance demands, as competitions elicit stronger psychophysiological reactions compared to training (Blascovich and Mendes, 2000). This dual-timepoint design enabled comparison of stress appraisals in low- and high-stress contexts to examine how HP influences athletes’ psychological responses. PHAS and ACSI-28 were not reapplied before competitions, as these scales measure relatively stable constructs such as parenting perceptions and general coping skills, whereas CTSS captures context-sensitive stress appraisals that vary between training and competitive settings. This dual-timepoint design enabled comparison of stress appraisals in low- and high-stress contexts to examine how HP influences athletes’ psychological responses. Data collection occurred in a controlled setting, with athletes completing surveys alone in the locker room before competitions to ensure privacy and minimize external influences. Trained researchers ensured confidentiality in controlled settings.

### 2.3 Measures

Perceived Helicopter Parenting Attitudes Scale (PHAS): This 25-item, four-point Likert scale (1 = strongly disagree, 4 = strongly agree), developed by Yılmaz (2019) for Turkish populations, assesses parenting attitudes across four subscales: Basic Trust and Life Skills (six items, e.g., “My parent tries to solve all my problems”), Emotional-Personal Life (six items, e.g., “My parent wants to be involved in every emotional decision I make”), Academic Life (six items, e.g., “My parent constantly monitors my school performance”), and Ethical-Moral (seven items, e.g., “My parent frequently reminds me of what is morally right or wrong”). Total scores range from 25 to 100, with scores of 56 or higher indicating helicopter parenting, 32–55 indicating regular parenting, and 21–31 indicating uninterested parenting, as established by Yılmaz (2019). Athletes completed the scale separately for mothers and fathers to account for potential differences in parental influence. Cronbach’s alpha for subscales ranged from 0.70 to 0.82, indicating acceptable reliability.

Athletic coping skills inventory-28 (ACSI-28): The ACSI-28 is a 28-item self-report scale originally developed by Smith et al. (1995). Adapted for Turkish populations by Özcan and Günay (2017), this 26-item, four-point Likert scale, ranging from 0 (almost never) to 3 (almost always), measures seven subscales: Coping with Adversity (four items, e.g., “I handle pressure well”), Concentration (four items, e.g., “I become easily distracted and lose focus during competition”), Coachability (four items, e.g., “I accept constructive criticism without getting upset”), Confidence and Achievement Motivation (three items, e.g., “I believe I can overcome any challenge to reach my goals”), Goal Setting/Mental Preparation (four items, e.g., “I set goals for myself and mentally prepare before competition”), Peaking Under Pressure (four items, e.g., “I perform at my best when it matters most”), and Freedom from Worry (three items, e.g., “I worry too much about performing poorly”), Two items in the Coachability subscale are reverse-scored. Total scores range from 0 to 78, with higher scores indicating stronger coping skills. Cronbach’s alpha for subscales ranged from 0.62 to 0.80, demonstrating adequate reliability. Although Concentration (0.68), Confidence and Achievement Motivation (0.65), and Freedom from Worry (0.62) subscales yielded Cronbach’s alpha coefficients slightly below the conventional 0.70 threshold, values above 0.60 are considered acceptable in adaptation studies, particularly for multidimensional constructs (DeVellis, 2016; Nunnally and Bernstein, 1994).

Challenge and Threat in Sport Scale (CTSS): Adapted by Gürbüz et al. (2021) for Turkish populations, this 11-item, five-point Likert scale, ranging from 1 (not at all) to 5 (completely), measures two subscales: Challenge with five items (e.g., “I feel confident about performing well”) and Threat with 6 items (e.g., “I worry about performing poorly”). Total scores range from 5 to 25 for Challenge and 6 to 30 for Threat, with higher scores indicating stronger respective appraisals. Cronbach’s alpha was 0.80 for Challenge and 0.84 for Threat, indicating high reliability.

### 2.4 Statistical methods

Data were analyzed using SPSS version 22.0. Normality was assessed using Kolmogorov-Smirnov and Shapiro-Wilk tests, revealing non-normal distributions for athletic coping skills, which prompted the use of Mann-Whitney *U*-tests to compare athletic coping skills between the HP and RP groups, aligning with the study’s objective to examine group differences in coping skills. To explore the linear relationship between perceived HP scores and athletic coping skills, Pearson correlation analyses were conducted, as suggested by prior studies indicating continuous relationships between parenting styles and psychological outcomes (Dorsch et al., 2016). Two 2 × 2 repeated-measures ANOVAs, with group (HP vs. RP) and time (training vs. competition) as factors, were used to examine differences in Challenge and Threat scores, addressing the study’s objective to investigate stress appraisals across contexts. Assumptions of sphericity and homogeneity of variance were verified using Mauchly’s test and Levene’s test, respectively. Statistical significance was set at *p* < 0.05 for all analyses.

## 3 Results

Demographic characteristics of 302 competitive youth athletes (mean age: 13.34 ± 0.60 years) showed 45% were female and 54% were male, with 78.2% participating in team sports and 21.8% in individual sports, as presented in Table 1. Most athletes, specifically 70.5%, self-selected their sport. Parental demographics (mothers: mean age 38.78 ± 5.34 years; fathers: 42.63 ± 5.60 years) showed 61.6% of mothers had primary/secondary education, and 35.8% of fathers were self-employed (Table 2). Of mothers, 31.4% (*n* = 125) were perceived as HP, 67.6% as RP, and 1% as uninterested; for fathers, 14.3% were HP, 81.4% as RP, and 4.3% as uninterested. Mothers scored higher on HP attitudes across all subscales (*p* < 0.001, Table 3).

**TABLE 1:** Demographic characteristics of young competitive athletes.

Variable	Category	*f* (%)
**Gender**	Female	136 (45)
	Male	166 (55)
**Branch**	Football	92 (30.5)
	Volleyball	59 (19.5)
	Handball	44 (14.6)
	Basketball	41 (13.6)
	Taekwondo	18 (6)
	Swimming	16 (5.3)
	Athletics	11 (3.6)
	Judo	7 (2.3)
	Table-tennis	5 (1.7)
	Futsal	3 (1)
	Wrestling	3 (1)
	Kickboxing	2 (0.7)
	Archery	1 (0.3)
**Reason for choosing the sport**	Self-decision	213 (70.5)
	Parents	44 (14.6)
	Teacher	45 (14.9)
	**Total**	302 (100)

Data represent the demographic characteristics of young competitive athletes (*n* = 302) participating in the study.

**TABLE 2:** Demographic characteristics of athletes’ parents.

Variable	Category	Mother	Father
		*f* (%)	*f* (%)
**Education level**	Primary school	89 (29.5)	73 (24.2)
	Secondary school	97 (32.1)	72 (23.8)
	High school	83 (27.5)	100 (33.1)
	University	33 (10.9)	57 (18.9)
**Occupation**	Housewife	243 (80.5)	–
	Farmer	–	14 (4.6)
	Worker	27 (8.9)	87 (28.8)
	Self-employment	11 (3.6)	108 (35.8)
	Tradesman	7 (2.3)	41 (13.6)
	Civil servant	6 (1.5)	39 (12.9)
	Unemployed	4 (1.3)	4 (1.3)
	Retired	4 (1.3)	9 (3)
	**Total**	302 (100)	302 (100)

Data represent the educational and occupational characteristics of parents of young competitive athletes (*n* = 302).

**TABLE 3:** Comparison of perceived helicopter parenting (HP) attitudes between mothers and fathers.

**Subscale**	**Mother**	**Father**		
	Median (min max)	Median (min max)	*Z*	*P*
Total HP	51 (22–84)	47 (21–70)	8.750	0.001*
BTLS	10 (6–24)	9 (6–20)	8.341	0.001*
EPL	11 (4–16)	10 (4–16)	7.870,50	0.001*
AL	13 (5–20)	11 (5–20)	7.274,50	0.001*
EM	17 (6–24)	17 (6–24)	12.909	0.001*

Wilcoxon signed rank test,

*p < 0.05. BTLS, Basic Trust and Life Skills; EPL, Emotional-Personal Life; AL, academic life; EM, Ethical-Moral. Although the median of mothers’ and fathers’ helicopter attitudes on ethical-moral issues was similar, their score distributions differed significantly.

### 3.1 Athletic coping skills

Among 302 athletes (HP: *n* = 51; RP: *n* = 251), those with HP parents had significantly lower total ACSI-28 scores (*M* = 44.55, SD = 10.04) compared to RP parents (*M* = 50.02, SD = 10.36; *U* = 4.471, *p* = 0.001). Subscale differences were significant for Coping with Adversity (*p* = 0.017), Concentration (*p* = 0.001), Confidence and Achievement Motivation (*p* = 0.024), Goal Setting/Mental Preparation (*p* = 0.001), Peaking Under Pressure (*p* = 0.001), and Freedom from Worry (*p* = 0.001), but not for Coachability (*p* = 0.119), as shown in Table 4. Exploratory Pearson correlations revealed negative associations between HP scores and total coping skills (mother *r* = −0.452; father *r* = −0.332 *p* < 0.001), supporting the group-based findings, as detailed in Table 5. These results align with the study’s objective to examine the relationship between perceived helicopter parenting and athletic coping skills, indicating that higher helicopter parenting is associated with reduced coping abilities in competitive youth athletes.

**TABLE 4:** Comparison of athletic coping skills inventory-28 (ACSI-28) scores between HP and RP groups.

Subscale	HP (M ± SD)	RP (M ± SD)	*U*	*P*
Coping with adversity	7.00 ± 2.76	7.92 ± 2.89	5.056	0.017*
Concentration	7.29 ± 2.36	8.63 ± 2.42	4.274	0.001*
Coachability	6.24 ± 1.96	6.76 ± 2.37	7.280	0.119
Confidence and achievement motivation	6.44 ± 2.03	7.08 ± 1.97	5.130	0.024*
Goal settings/mental preparation	6.94 ± 2.52	8.43 ± 2.54	4.309	0.001*
Peaking under pressure	5.47 ± 3.04	7.35 ± 2.60	4.148	0.001*
Freedom from worry	4.37 ± 2.32	5.66 ± 2.11	5.779	0.001*
Total ACSI-28	44.55 ± 10.04	50.02 ± 10.36	4.471	0.001*

Mann-Whitney *U*-test

*p < 0.05, HP, helicopter parenting; RP, regular parenting.

**TABLE 5:** Correlations between maternal and paternal helicopter parenting, athletic coping skills, and stress appraisals.

Variable		PHAS mother	PHAS father	ACSI total	CTSS challenge competition	CTSS threat competition
PHAS mother	r	—	0.332	−0.452	−0.447	0.389
	*p*		0.008*	0.004*	0.011*	0.002*
PHAS father	r		—	−0.395	−0.410	0.328
	*p*			0.001*	0.005*	0.001*
ACSI total	r			—	0.452	−0.391
	*p*				0.004*	0.016*
CTSS challenge competition	r				—	−0.459
	*p*					0.001*

The “r” values represent Pearson correlation coefficients,

*p < 0.05.

### 3.2 Challenge and threat states

No group differences emerged in challenge (HP: *M* = 20.12, SD = 3.57; RP: *M* = 20.64, SD = 3.28; *p* = 0.327) or threat (HP: *M* = 20.10, SD = 5.09; RP: *M* = 19.28, SD = 5.31; *p* = 0.404) states during training. Before competitions, HP athletes showed reduced challenge (*M* = 17.21, SD = 5.66 vs. RP: *M* = 20.04, SD = 3.79; *p* < 0.001) and increased threat (*M* = 22.59, SD = 4.45 vs. RP: *M* = 17.77, SD = 5.44; *p* = 0.001; Table 6). Repeated-measures ANOVA revealed significant Group × Time interactions for challenge [F(1,300) = 7.88, *p* = 0.005, η^2^ = 0.013] and threat [F(1,300) = 12.18, *p* < 0.001, η^2^ = 0.020; Table 7].

**TABLE 6:** Challenge and threat scores by parenting group across training and competition.

Stress appraisals	Parenting	Training			Competition		
		M ± SD	*U*	*P*	M ± SD	*U*	*P*
Challenge	HP	20.12 ± 3.57	5.885	0.327	17.21 ± 5.66	3.309	< 0.001*
	RP	20.64 ± 3.28			20.04 ± 3.79		
Threat	HP	20.10 ± 5.09	6.872	0.404	22.59 ± 4.45	8.209	0.001*
	RP	19.28 ± 5.31			17.77 ± 5.44		

Mann-Whitney *U*-test,

*p < 0.05, HP, helicopter parenting; RP, regular parenting.

**TABLE 7:** Two way repeated-measures ANOVA results.

Source	Challenge			Threat		
	F (1, 300)	*P*	η^2^p	F (1, 300)	*P*	η^2^p
Parenting group (helicopter vs. regular)	16.695	0.001*	0.027	24.116	0.001*	0.039
Test time (training vs. competition)	18.283	0.001*	0.030	0.724	0.395	0.001
Parenting attitude*time interaction	7.882	0.005*	0.013	12.179	0.001*	0.020

Results from a two-way repeated-measures ANOVA comparing challenge and threat appraisals by parenting group (helicopter vs regular) and test time (training vs. competition) (*n* = 302).

*p < 0.05.

## 4 Discussion

This study is the first to examine the relationship between perceived HP and athletic coping skills, as well as challenge and threat appraisals, among Turkish competitive youth athletes aged between 13 and 15 years. The findings indicate that athletes with parents exhibiting HP exhibit lower scores on athletic coping skills, except for coachability, and experience increased threat and reduced challenge appraisals before competitions, but not during training. These results align with SDT, which posits that overcontrolling parenting behaviors may undermine athletes’ autonomy, limiting their ability to manage competitive stress effectively (Deci and Ryan, 2000; Ryan and Deci, 2017). The lack of group differences in coachability may reflect coaches’ role in maintaining consistent training interactions, regardless of parenting style, as suggested by Holt et al. (2008).

The higher prevalence of HP among mothers (31.4%) compared to fathers (14.3%) reflects cultural norms in Turkey, where mothers, often housewives (79.4%), assume primary caregiving roles (Yılmaz, 2020). This contrasts with Western studies reporting gender-balanced HP (Segrin et al., 2012), underscoring the importance of culturally sensitive research. Recent literature highlights the bidirectional nature of parent-athlete relationships, suggesting that the quality of parental involvement, rather than quantity, shapes psychological outcomes (Dorsch et al., 2016). The biopsychosocial model of stress explains the increased threat appraisals in athletes with parents exhibiting HP, as excessive parental control may heighten perceived competetive demands, limiting coping resources (Blascovich and Mendes, 2000; Gürbüz et al., 2021). Furthermore, Pepe et al. (2025) found that positive thinking enhances challenge appraisals in Turkish athletes, suggesting cognitive strategies could counteract the negative effects of helicopter parenting on stress appraisals.

The negative association between helicopter parenting and athletic coping skills, measured by the ACSI-28 highlights specific deficits in coping with adversity, concentration, goal setting, peaking under pressure, and freedom from worry among athletes with parents exhibiting helicopter parenting. These findings suggest that excessive parental involvement may limit athletes’ opportunities to develop independent coping strategies, aligning with prior research indicating that overcontrolling parenting hinders psychological development (Padilla-Walker and Nelson, 2012; LeMoyne and Buchanan, 2011). The significant Group × Time interactions for challenge and threat appraisals, measured by the CTSS indicate that helicopter parenting’s impact is context-specific, emerging primarily in high-stakes competitive settings rather than training.

These findings have practical implications such that coaches and sports programs could educate parents on balanced involvement, emphasizing encouragement without overcontrol, to support athletes’ autonomy (Ryan and Deci, 2017). Community workshops targeting Turkish mothers could address cultural tendencies toward HP. Future research should adopt longitudinal designs, incorporate parental self-reports, and include biophysical measures (e.g., heart rate variability) to validate stress appraisals. Cross-cultural comparisons could further clarify HP’s effects across diverse contexts.

### 4.1 Limitations

This study has several limitations. The cross-sectional design precludes causal inferences about the relationship between helicopter parenting, athletic coping skills, and stress appraisals. Reliance on athletes’ perceptions may introduce self-report bias, as parental behaviors were not assessed via self-reports or expert observations. The sample, drawn from Turkish youth athletes, may not generalize to other cultural or age groups due to unique parenting norms (Yılmaz, 2020). The absence of biophysical measures limits validation of challenge/threat states. Additionally, the group creation procedure, which excluded athletes with mixed parental attitude such as one parent HP and one RP or uninterested parenting attitudes to ensure clear comparisons, may oversimplify the complexity of parental influences, as real-world parenting dynamics often involve mixed behaviors. This exclusion reduced the sample size from 398 to 302, enhancing group comparison clarity but potentially limiting the representation of diverse parenting dynamics. Furthermore, in the Turkish version of the ACSI-28 scale, Concentration (α = 0.68), Confidence and Achievement Motivation (α = 0.65), and Freedom from Worry (α = 0.62) subscales had Cronbach’s alpha values below 0.70, indicating moderate reliability that may affect measurement precision for these subscales. Finally, academic or non-sport outcomes were not assessed, which could provide a broader understanding of HP’s effects.

## 5 Conclusion

Perceived helicopter parenting is associated with lower athletic coping skills and increased threat appraisals before competitions in Turkish youth athletes. These findings highlight the importance of balanced parental involvement to support athletes’ psychological skills. Community programs targeting Turkish parents, particularly mothers, could foster encouragement without overcontrol. Longitudinal research and cross-cultural studies are needed to further elucidate HP’s impact on youth sports.

## Data Availability

The data sets analyzed during the current study are available from the corresponding authors on reasonable request. Data will be shared without personal information of participants.
